# Metabolic syndrome and epigenetic aging: a twin study

**DOI:** 10.1038/s41366-024-01466-x

**Published:** 2024-01-25

**Authors:** Tiina Föhr, Arne Hendrix, Anna Kankaanpää, Eija K. Laakkonen, Urho Kujala, Kirsi H. Pietiläinen, Terho Lehtimäki, Mika Kähönen, Olli Raitakari, Xiaoling Wang, Jaakko Kaprio, Miina Ollikainen, Elina Sillanpää

**Affiliations:** 1 Faculty of Sport and Health Sciences, Gerontology Research Center, University of Jyväskylä, Jyväskylä, Finland; 2 Physical Activity, Sport & Health Research Group, Department of Movement Sciences, KU Leuven – University of Leuven, Leuven, Belgium; 3 Obesity Research Unit, Research Program for Clinical and Molecular Metabolism, Faculty of Medicine, University of Helsinki, Helsinki, Finland; 4 Healthy Weight Hub, Endocrinology, Abdominal Center, Helsinki University Hospital, University of Helsinki, Helsinki, Finland; 5 Department of Clinical Chemistry, Fimlab Laboratories, and Finnish Cardiovascular Research Center – Tampere, Faculty of Medicine and Health Technology, Tampere University, Tampere, Finland; 6 Department of Clinical Physiology, Tampere University Hospital, and Finnish Cardiovascular Research Center – Tampere, Faculty of Medicine and Health Technology, Tampere University, Tampere, Finland; 7 Centre for Population Health Research, University of Turku and Turku University Hospital, Turku, Finland; 8 Research Centre of Applied and Preventive Cardiovascular Medicine, University of Turku, Turku, Finland; 9 Department of Clinical Physiology and Nuclear Medicine, Turku University Hospital, Turku, Finland; 10 Georgia Prevention Institute (GPI), Medical College of Georgia, Augusta University, Augusta, GA USA; 11 Institute for Molecular Medicine Finland (FIMM), University of Helsinki, Helsinki, Finland; 12 Minerva Foundation Institute for Medical Research, Helsinki, Finland; 13 The Wellbeing Services County of Central Finland, Jyväskylä, Finland

**Keywords:** Signs and symptoms, Genetics

## Abstract

**Background:**

Metabolic syndrome (MetS) is associated with premature aging, but whether this association is driven by genetic or lifestyle factors remains unclear.

**Methods:**

Two independent discovery cohorts, consisting of twins and unrelated individuals, were examined (*N* = 268, aged 23–69 years). The findings were replicated in two cohorts from the same base population. One consisted of unrelated individuals (*N* = 1 564), and the other of twins (*N* = 293). Participants’ epigenetic age, estimated using blood DNA methylation data, was determined using the epigenetic clocks GrimAge and DunedinPACE. The individual-level linear regression models for investigating the associations of MetS and its components with epigenetic aging were followed by within-twin-pair analyses using fixed-effects regression models to account for genetic factors.

**Results:**

In individual-level analyses, GrimAge age acceleration was higher among participants with MetS (*N* = 56) compared to participants without MetS (*N* = 212) (mean 2.078 [95% CI = 0.996,3.160] years vs. −0.549 [−1.053,−0.045] years, between-group *p* = 3.5E-5). Likewise, the DunedinPACE estimate was higher among the participants with MetS compared to the participants without MetS (1.032 [1.002,1.063] years/calendar year vs. 0.911 [0.896,0.927] years/calendar year, *p* = 4.8E-11). An adverse profile in terms of specific MetS components was associated with accelerated aging. However, adjustments for lifestyle attenuated these associations; nevertheless, for DunedinPACE, they remained statistically significant. The within-twin-pair analyses suggested that genetics explains these associations fully for GrimAge and partly for DunedinPACE. The replication analyses provided additional evidence that the association between MetS components and accelerated aging is independent of the lifestyle factors considered in this study, however, suggesting that genetics is a significant confounder in this association.

**Conclusions:**

The results of this study suggests that MetS is associated with accelerated epigenetic aging, independent of physical activity, smoking or alcohol consumption, and that the association may be explained by genetics.

## Introduction

Metabolic syndrome (MetS) is a significant precursor to cardiovascular diseases and type 2 diabetes [1]. MetS refers to the co-occurrence of several known cardiovascular risk factors that typically increase with age, such as insulin resistance, obesity, atherogenic dyslipidemia, and hypertension [2, 3]. The worldwide prevalence of adulthood MetS is approximately 30–40% [4, 5]. MetS is strongly linked to a lifestyle characterized by an unhealthy diet and physical inactivity [6], and it may lead to premature aging [7–9]. However, it is unclear whether the accumulation of MetS components increase the likelihood of developing diseases that shorten lifespan or if the accumulation of MetS components itself accelerates the aging process.

Epigenetics, particularly age-related changes in DNA methylation (DNAm), constitute the primary hallmark of biological aging [10, 11]. Epigenetic mechanisms regulate gene expression and help us adapt to different environments and lifestyles, including unhealthy diet and physical inactivity, which are associated with the increasing prevalence of MetS. Genome-wide DNAm data can be used to construct composite scores, i.e. epigenetic clocks, which provide an estimate of an individual’s biological age. Epigenetic clocks are algorithms that aim to quantify biological aging using DNAm levels at specific CpG sites. Epigenetic clocks summarize the effects of genetic susceptibility, as well as the cumulative effect of lifestyle and environmental factors, on physiological aging over the life course [12, 13].

The epigenetic clock GrimAge was developed to predict mortality [14]. Compared to previously developed clocks, GrimAge may best capture the DNAm changes associated with MetS and its components [9, 15]. The recently developed DunedinPACE estimator differs from GrimAge and other predecessors because it has been developed to predict the pace of aging measured over a 20-year follow-up. DunedinPACE operationalizes aging as a decline in physiological integrity over the years [16] and may, therefore, be a particularly good marker for assessing the effects of the age-related accumulation of MetS risk factors on epigenetic aging.

The epigenome is an intriguing target for both MetS and age-related physiological changes because it is a major determinant of gene expression that is modifiable by the environment and lifestyle. A more adverse metabolic risk profile, or MetS, is associated with accelerated epigenetic aging [8, 9, 15, 17–19]. However, the results vary by epigenetic clock, and to the best of our knowledge, no previous study has investigated the association of MetS with the most recent clock, DunedinPACE, and/or considered the effects of genetic factors. Genotype has an important effect on both the components of MetS and the epigenome [20], which means genetic confounding is possible when assessing the association between MetS and epigenetic aging. Thus, our objective was to investigate the cross-sectional association of MetS and its components with epigenetic aging. We employed two recent epigenetic clocks, GrimAge and DunedinPACE, in our analyses. To control for genotype and sex, age, and early childhood environmental factors shared by twin siblings, we employed within-twin-pair comparisons.

## Methods

### Study populations

The data (*N* = 268, 57% female) for the primary, discovery-oriented analyses of this study were drawn from two Finnish population-based cohort studies: the Finnish Twin Cohort (FTC) [21–24] and the Estrogenic Regulation of Muscle Apoptosis (ERMA) study [25] (for details see Supplement 1). The age range of the pooled study population covered most adulthood, from 23 to 69 years. Those who fulfilled the criteria for having MetS constituted 21% of the participants.

### Replication analyses

To validate our primary results, we replicated the analyses using two cohorts (Supplement 1). The individual-level analyses were replicated in a large, independent Finnish cohort study, The Young Finns Study (YFS) [26, 27], which consisted of 1 564 unrelated individuals (55% female) aged 34–49 years and of which 22% had MetS. The within-twin-pair analyses were replicated in a dataset of Essential Hypertension Epigenetics Study (EH-Epi) [23, 28], which consisted of 293 twins (61% female) aged 56–69 years and of which 32% had MetS.

### Research ethics

Previously given consents covered our study (Supplement 2).

### Epigenetic aging

Blood-based DNAm profiles were obtained using Illumina’s Infinium HumanMethylation450 BeadChip or the Infinium MethylationEPIC BeadChip (Illumina, San Diego, CA, USA). In our previous articles, we described the generation, preprocessing, and normalization of DNAm data [13, 29]. In this study’s analyses, we used the epigenetic clocks GrimAge [14] and DunedinPACE [16]. Recently, epigenetic clocks based on principal components (PCs) have been developed to bolster the reliability and validity of the clocks [30]. We produced PC-based GrimAge estimates using an R package (https://github.com/MorganLevineLab/PC-Clocks). Age acceleration in years (GrimAgeAA) was defined as the residual obtained from regressing the estimated epigenetic age on chronological age. In addition, we obtained PC-based GrimAge components (adjusted for age), including DNAm smoking pack–years, DNAm ADM, DNAm B2M, DNAm cystatin C, DNAm GDF15, DNAm leptin, DNAm PAI-1, and DNAm TIMP-1. DunedinPACE provided an estimate of the pace of aging in years per calendar year [16]. DunedinPACE was calculated using a publicly available R package (https://github.com/danbelsky/DunedinPACE).

### Metabolic syndrome

MetS was determined according to the National Cholesterol Education Program (NCEP) Adult Treatment Panel III (ATP III) [31], which was updated by the American Heart Association and the National Heart Lung and Blood Institute in 2005 [32].

### Components of metabolic syndrome

*Waist circumference* was measured at the midpoint between the lowest rib and the iliac crest by trained research nurses. *Fasting high density lipoprotein (HDL) cholesterol, triglyceride, and fasting glucose levels* were measured via blood samples taken from the participants after an overnight fast. *Blood pressure* was measured with a sphygmomanometer. Systolic and diastolic blood pressure were measured three or two times (for the FTC and ERMA, respectively), and the mean of these measurements was used in our analysis. More detailed information about the measurement methods used has been presented previously for the FTC [21–23] and ERMA [25, 33]. The use of cholesterol- and glucose-lowering medications as well as of antihypertensives was self-reported with brand names and confirmed by a physician or a nurse during a medical examination.

### Other covariates

*Alcohol consumption* was calculated as the number of alcoholic drinks (1 drink = 12 g ethanol) consumed per week. *Smoking* was classified according to the following three categories: never, former, and current smoker. Current smokers included both daily and occasional smokers.

*Physical activity*. In the FTC, the Baecke questionnaire was used to assess physical activity [34]. Following three indexes; the work index, the sport index, and the leisure-time index, were calculated using 16 items. All responses were given on a five-point scale except for questions regarding the main occupation and the types of two main sports. In the original publication, the test–retest reliability scores of the work, sport, and leisure-time indices were 0.88, 0.81, and 0.74, respectively [34]. The Baecke questionnaire has been validated for cardiorespiratory fitness among Finnish twins [35]. For the analysis, the participants were divided into the following three groups of physical activity according to the sport index: low (Groups 1–2), medium (Group 3), and high physical activity (Groups 4–5).

For the ERMA study, the participants’ self-reported physical activity was measured using a single-question scale that included seven physical-activity-level categories ranging from necessary daily activities and routines to participation in competitive sports [36]. For the analysis, the participants were further divided into the groups of low (Groups 1–2), medium (Groups 3–4), and high physical activity (Groups 5–7). The test–retest reliability, concurrent validity against accelerometer-measured physical activity, and associations with several physical performance measurements have been reported previously [37].

### Statistical analysis

We analyzed differences in epigenetic aging (age acceleration/pace of aging) between the participants with and without MetS using linear regression analyses adjusted for the within-pair dependency of twins (family relatedness), age, and sex (including the interaction term age*sex) (Release 16; Stata Corporation, College Station, TX, USA). In addition, we employed a linear regression analysis to assess the association between specific MetS components and epigenetic aging. The dependent variable was age acceleration/pace of aging, while the independent variable was one of the MetS components (waist circumference, HDL cholesterol, triglycerides, fasting glucose, systolic blood pressure, and diastolic blood pressure). For triglycerides and fasting glucose, a natural log transformation was performed due to the skewed distribution of the variables. Model 1 included an adjustment for the family relatedness, age, and sex (including an interaction term age*sex). Then, we carried out the analyses with multivariable adjustments. We adjusted Model 1 for smoking status, alcohol consumption, and physical activity level (Model 2). Finally, we adjusted Model 2 for medications (cholesterol, blood pressure, and blood glucose; Model 2 + medications). After the individual-level analyses, fixed-effects within-twin-pair regression models were conducted for all twin pairs, as well as separately for the monozygotic (MZ) and dizygotic (DZ) pairs. If an association between MetS components and accelerated epigenetic aging is observed in the co-twin control design, particularly in the MZ pairs, this provides strong evidence for an association between MetS and epigenetic aging, independent of the genetic and other shared effects. We present exact two-sided *p* values and set the nominal level of significance at *p* ≤ .05.

## Results

### Participant characteristics

The mean age of the participants was 40.0 years (SD 14.6). The correlation between chronological age and DNAm GrimAge (mean 53.1, SD 12.5) was 0.95, while the correlation with DunedinPACE was 0.40. Furthermore, age acceleration (GrimAgeAA) exhibited a correlation of 0.61 with the pace of aging (DunedinPACE). The characteristics of the study participants, stratified by MetS status, are presented in Table 1. In total, 56 participants (21%) met the criteria for having MetS, with 59% being women. The mean age of the participants with MetS was 52.6 years (SD 15.6), ranging from 23 to 69 years.

**Table 1:** Descriptive characteristics of the participants by MetS status: Presentation of MetS component characteristics separately for all, female, and male participants.

Characteristic	Participants without MetS (*N* = 212)	Participants with MetS (*N* = 56)
*Sex, N (%) of participants*		
Female	121 (57.1)	33 (58.9)
Male	91 (42.9)	23 (41.1)
*Age, mean (SD) range, years*	36.7 (12.3) 23–69	52.6 (15.6) 23–69
*Cigarette smoking, N (%) of participants*		
Never smokers	108 (50.9)	23 (41.1)
Former smokers	43 (20.3)	18 (32.1)
Current smokers	60 (28.3)	14 (25.0)
*Alcohol, mean (SD), drinks per week*^*∞*^	4.6 (6.1)	5.1 (6.9)
*Level of physical activity, N (%) of participants*		
Low	60 (28.3)	25 (44.6)
Medium	63 (29.7)	19 (33.9)
High	79 (37.3)	7 (12.5)
*Body mass index, mean (SD), kg/m*^*2*^	26.1 (5.1)	31.9 (5.3)
*Components of MetS, mean (SD)*		
Waist Circumference (cm)		
All	88.0 (12.5)	106.7 (12.7)
Female	85.2 (13.1)	103.4 (12.9)
Male	91.4 (11.2)	111.4 (11.3)
HDL cholesterol (mmol/l)		
All	1.7 (0.4)	1.4 (0.5)
Female	1.8 (0.5)	1.5 (0.5)
Male	1.5 (0.4)	1.2 (0.4)
Triglycerides (mmol/l)		
All	0.9 (0.5)	1.6 (1.0)
Female	0.9 (0.5)	1.4 (0.6)
Male	0.9 (0.5)	1.8 (1.3)
Fasting glucose (mmol/l)		
All	5.0 (0.5)	6.2 (1.6)
Female	5.0 (0.5)	6.0 (1.0)
Male	5.1 (0.5)	6.5 (2.2)
Blood pressure (mmHg)		
Systolic		
All	128.0 (17.2)	143.5 (18.1)
Female	126.5 (19.0)	142.7 (19.7)
Male	130.5 (14.4)	145.5 (17.1)
Diastolic		
All	74.6 (12.1)	86.0 (9.6)
Female	75.7 (12.1)	86.0 (10.2)
Male	73.8 (12.5)	87.6 (8.4)
*Medication, N (%) of participants*		
Cholestero	0 (0)	7 (12.5)
Hypertension	14 (6.6)	17 (30.4)
Glucose	0 (0)	4 (7.1)

^∞^one drink = 12 g/100% alcohol.

DNAm, *DNA* methylation; *HDL* high-density lipoprotein; *MetS* metabolic syndrome.

### Differences in epigenetic aging according to MetS status: individual-level analyses

**Fig. 1:**
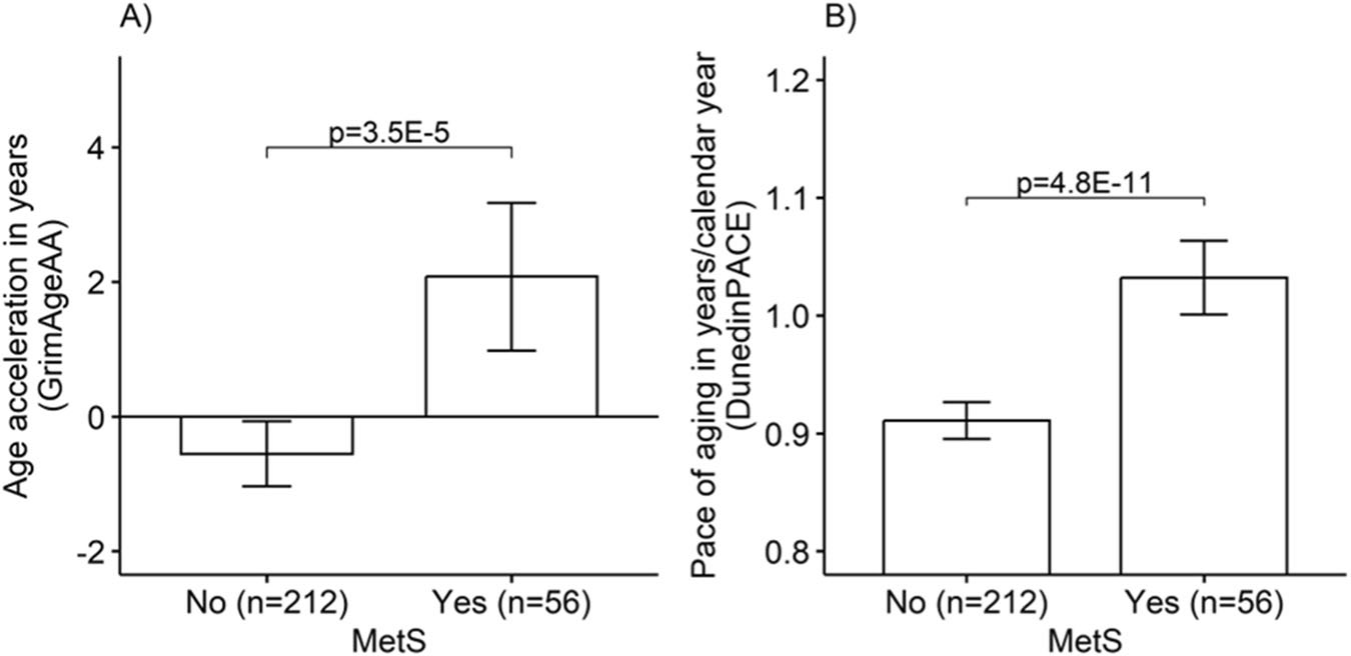
Differences in epigenetic aging according to the status of metabolic syndrome (MetS). Means and 95% confidence intervals of **A** age acceleration (GrimAgeAA) and **B** the pace of aging (DunedinPACE). Note: Adjusted for family relatedness, age, sex, and age*sex interaction.

Figure 1 presents the differences in age acceleration/pace of aging by MetS status for all participants. GrimAgeAA was higher among participants with MetS (*n* = 56) compared to participants without MetS (*n* = 212) (mean 2.078 [95% CI = 0.996, 3.160] years vs. −0.549 [−1.053, −0.045] years, between-group *p* = 3.5E-5). Likewise, the DunedinPACE estimate was higher among the participants with MetS compared to the participants without MetS (1.032 [1.002, 1.063] years/calendar year vs. 0.911 [0.896, 0.927] years/calendar year, *p* = 4.8E-11) (Supplementary Table 1).

### Association between MetS components and epigenetic aging: individual-level analyses

An adverse profile of MetS components was associated with accelerated epigenetic aging. In Model 1 (adjusted for age and sex), all MetS components except for blood pressure were associated with GrimAgeAA. More specifically, greater waist circumference (standardized regression coefficient β = 0.235, *p* = 2.6E-4), higher levels of triglycerides (0.218, *p* = 2.6E-4) and fasting glucose (0.163, *p* = .027), and a lower level of HDL cholesterol (−0.231, *p* = .001) were associated with higher GrimAgeAA. Further adjustments for lifestyle factors (Model 2) and medication (Model 2 + medication) attenuated these associations to nonsignificant levels (Table 2).

**Table 2:** The association between metabolic syndrome components and age acceleration in years (GrimAgeAA).

	Model 1			Model 2			Model 2 + medication		
	*β*	b (95% CI)	*p*-value	*β*	b (95% CI)	*p*-value	*β*	b (95% CI)	*p*-value
**Waist circumference (cm)**	0.235	0.060 (0.028, 0.091)	2.6E-4	0.092	0.023 (−0.009, 0.054)	0.154			
**HDL cholesterol (mmol/L)**	−0.231	−1.919 (−1.156, −0.006)	0.001	−0.076	−0.631 (−1.558, 0.296)	0.181	−0.076	−0.631 (−1.560, 0.298)	0.182 ^µ^
**Triglycerides (mmol/L)**^**∞**^	0.218	1.660 (−2.947, 3.042)	2.6E-4	0.081	0.618 (−0.185, 1.421)	0.130	0.081	0.618 (−0.187, 1.423)	0.131^µ^
**Fasting glucose (mmol/L)**^**∞**^	0.163	4.946 (0.567, 9.326)	0.027	0.062	1.830 (−1.615, 5.274)	0.296	0.062	1.832 (−1.617, 5.282)	0.296^α^
**Systolic bp (mmHG)**	−0.001	−0.000 (−0.027, 0.027)	0.990	−0.008	−0.002 (−0.024, 0.021)	0.890	−0.010	−0.002 (−0.024, 0.021)	0.868^π^
**Diastolic bp (mmHG)**	0.095	0.029 (−0.016, 0.074)	0.209	0.021	0.006 (−0.033, 0.045)	0.748	0.020	0.006 (−0.034, 0.045)	0.772^π^

Model 1 adjusted for family relatedness, age and sex, (age*sex).

Model 2 adjusted for family relatedness, age, sex, (age*sex), smoking status, alcohol consumption and physical activity.

*HDL*, high-density lipoprotein, *bp*, blood pressure.

^µ^use of cholesterol lowering medications, ^α^use of blood glucose lowering medications, ^π^use of antihypertensives, ^∞^natural log transformation was performed due to skewed distribution of variable.

One sample excluded for extreme log-transformed fasting glucose (mmol/L) value of over 2.75.

*β* standardized regression coefficient, *b* unstandardized regression coefficient, *CI* confidence interval.

In Model 1, all MetS components except for systolic blood pressure were associated with the DunedinPACE estimate. Greater waist circumference (0.349, *p* = 1.0E-7), higher levels of triglycerides (0.255, *p* = 3.1E-5), fasting glucose (0.264, *p* = 2.5E-4), and diastolic blood pressure (0.171, *p* = .017), and a lower level of HDL cholesterol (−0.296, *p* = 1.3E-6) were associated with higher DunedinPACE estimates. Further adjustments for lifestyle factors (Model 2) and medication (Model 2 + medication) attenuated these associations, which, however, remained statistically significant (Table 3).

**Table 3:** The association between metabolic syndrome components and the pace of aging in years/calendar year (DunedinPACE).

	Model 1			Model 2			Model 2 + medication		
	*β*	b (95% CI)	*p*-value	*β*	b (95% CI)	*p*-value	*β*	b (95% CI)	*p*-value
**Waist circumference (cm)**	0.349	0.003 (0.002, 0.004)	1.0E-7	0.223	0.002 (0.001, 0.003)	0.001			
**HDL cholesterol (mmol/L)**	−0.296	−0.081 (−0.113, −0.049)	1.3E-6	−0.190	−0.054 (−0.089, −0.019)	0.003	−0.189	−0.054 (−0.089, −0.019)	0.003 ^µ^
**Triglycerides (mmol/L)**^**∞**^	0.255	0.064 (0.035, 0.094)	3.1E-5	0.163	0.042 (0.011, 0.074)	0.009	0.163	0.042 (0.011, 0.074)	0.009 ^µ^
**Fasting glucose (mmol/L)**^**∞**^	0.264	0.263 (0.124, 0.402)	2.5E-4	0.165	0.164 (0.040, 0.289)	0.010	0.163	0.162 (0.037, 0.287)	0.011^α^
**Systolic bp (mmHG)**	0.058	0.000 (−0.001, 0.001)	0.413	0.080	0.001 (−0.000, 0.001)	0.208	0.083	0.001 (−0.000, 0.001)	0.196^π^
**Diastolic bp (mmHG)**	0.171	0.002 (0.000, 0.003)	0.017	0.137	0.001 (0.000, 0.003)	0.046	0.140	0.001 (0.000, 0.003)	0.043^π^

Model 1 adjusted for family relatedness, age and sex.

Model 2 adjusted for family relatedness, age, sex, smoking status, alcohol consumption and physical activity.

*HDL*, high-density lipoprotein, *bp*, blood pressure.

^µ^use of cholesterol lowering medications, ^α^use of blood glucose lowering medications, ^π^use of antihypertensives, ^∞^natural log transformation was performed due to skewed distribution of variable.

One sample excluded for extreme log-transformed fasting glucose (mmol/L) value of over 2.75.

*β* standardized regression coefficient, *b* unstandardized regression coefficient, *CI* confidence interval.

### Association between MetS components and epigenetic aging: within-twin-pair analyses

The results of the fixed-effects within-twin-pair regression analyses are presented in Table 4 for GrimAge and in Table 5 for DunedinPACE. For all twin pairs (Table 4a), the specific MetS components were not associated with GrimAgeAA. However, an adverse profile in terms of MetS components (except for fasting glucose and systolic blood pressure) was associated with an accelerated pace of aging (DunedinPACE) (Table 5a). More specifically, in the base model (naturally adjusted for age and sex), greater waist circumference (unstandardized regression coefficient *β* = 0.002, *p* = .004), higher levels of triglycerides (0.048, *p* = .007), diastolic blood pressure (0.003, *p* = .005), and a lower level of HDL cholesterol (−0.064, *p* = .007) were associated with a higher DunedinPACE estimate. After further adjustments for lifestyle factors and medication, these associations were attenuated to nonsignificant levels (except for triglycerides).

**Table 4:** The association between metabolic syndrome components and age acceleration in years (GrimAgeAA): Within-twin-pair analyses, (a) all pairs, (b) monozygotic twin pairs, and (c) dizygotic twin pairs.

	Base model			Adjusted model*			Adjusted model* + medication		
	*β*	95% CI	*p*-value	*β*	95% CI	*p*-value	*β*	95% CI	*p*-value
**(a) All pairs (*N*** = **99)**									
Waist circumference (cm)	−0.002	(−0.049, 0.045)	0.931	−0.012	(−0.055, 0.031)	0.578			
HDL cholesterol (mmol/L)	−0.656	(−2.420, 1.108)	0.462	0.302	(−1.479, 2.083)	0.737	0.270	(−1.507, 2.047)	0.763 ^µ^
Triglycerides (mmol/L)^∞^	1.189	(−0.139, 2.516)	0.079	0.559	(−0.678, 1.795)	0.371	0.538	(−0.695, 1.772)	0.388 ^µ^
Fasting glucose (mmol/L)^∞^	−1.929	(−7.625, 3.766)	0.503	−1.232	(−6.181, 3.717)	0.621	−1.482	(−6.431, 3.467)	0.552^α^
Systolic bp (mmHG)	−0.001	(−0.048, 0.045)	0.949	−0.010	(−0.048, 0.028)	0.590	−0.011	(−0.049, 0.027)	0.571^π^
Diastolic bp (mmHG)	0.026	(−0.041, 0.094)	0.436	−0.003	(−0.070, 0.063)	0.925	−0.003	(−0.070, 0.064)	0.926^π^
**(b) Monozygotic twin pairs (*N*** = **62)**									
Waist circumference (cm)	−0.012	(−0.070, 0.046)	0.686	0.024	(−0.033, 0.081)	0.401			
HDL cholesterol (mmol/L)	−1.162	(−3.168, 0.844)	0.251	−0.041	(−2.077, 1.994)	0.968	−0.014	(−2.020, 1.991)	0.989 ^µ^
Triglycerides (mmol/L)^∞^	0.831	(−0.951, 2.614)	0.355	0.520	(−1.258, 2.297)	0.560	0.386	(−1.377, 2.148)	0.662 ^µ^
Fasting glucose (mmol/L)^∞^	−1.486	(−9.953, 6.980)	0.726	5.710	(−2.724, 14.144)	0.179	5.517	(−2.872, 13.907)	0.191^α^
Systolic bp (mmHG)	0.012	(−0.040, 0.065)	0.641	0.001	(−0.045, 0.046)	0.980	0.001	(−0.046, 0.048)	0.973^π^
Diastolic bp (mmHG)	−0.006	(−0.075, 0.063)	0.855	−0.001	(−0.077, 0.076)	0.986	−0.001	(−0.078, 0.077)	0.989^π^
**(c) Dizygotic twin pairs (*N*** = **37)**									
Waist circumference (cm)	0.006	(−0.075, 0.087)	0.878	−0.054	(−0.124, 0.017)	0.128			
HDL cholesterol (mmol/L)	−0.078	(−3.375, 3.218)	0.962	2.650	(−1.024, 6.324)	0.149	2.436	(−1.377, 6.249)	0.199 ^µ^
Triglycerides (mmol/L)^∞^	1.423	(−0.734, 3.579)	0.189	0.578	(−1.368, 2.524)	0.545	0.575	(−1.388, 2.538)	0.550 ^µ^
Fasting glucose (mmol/L)^∞^	−2.132	(−10.750, 6.487)	0.619	−3.206	(−10.063, 3.651)	0.343	−3.649	(−10.588, 3.289)	0.287^α^
Systolic bp (mmHG)	−0.019	(−0.107, 0.070)	0.670	−0.038	(−0.109, 0.033)	0.279	−0.029	(−0.102, 0.043)	0.411^π^
Diastolic bp (mmHG)	0.094	(−0.052, 0.239)	0.201	−0.075	(−0.214, 0.064)	0.276	−0.060	(−0.200, 0.080)	0.383^π^

*adjusted for smoking status, alcohol consumption, physical activity.

*HDL* high-density lipoprotein, *bp* blood pressure.

^µ^use of cholesterol lowering medications, ^α^use of blood glucose lowering medications, ^π^use of antihypertensives, ^∞^natural log transformation was performed due to skewed distribution of variable.

One sample excluded for extreme log-transformed fasting glucose (mmol/L) value of over 2.75.

*β* unstandardized regression coefficient, *CI* confidence interval.

**Table 5:** The association between metabolic syndrome components and the pace of aging in years/calendar year (DunedinPACE): Within-twin-pair analyses, (a) all pairs, (b) monozygotic twin pairs, and (c) dizygotic twin pairs.

	Base model			Adjusted model*			Adjusted model* + medication		
	*β*	95% CI	*p*-value	*β*	95% CI	*p*-value	*β*	95% CI	*p*-value
**(a) All pairs (*N*** = **99)**									
Waist circumference (cm)	0.002	(0.001, 0.003)	0.004	0.001	(−0.000, 0.002)	0.081			
HDL cholesterol (mmol/L)	−0.064	(−0.110, −0.018)	0.007	−0.043	(−0.094, 0.009)	0.106	−0.044	(−0.095, 0.007)	0.089 ^µ^
Triglycerides (mmol/L)^∞^	0.048	(0.013, 0.083)	0.007	0.038	(0.002, 0.074)	0.038	0.037	(0.002, 0.072)	0.039 ^µ^
Fasting glucose (mmol/L)^∞^	0.067	(−0.088, 0.223)	0.390	0.010	(−0.137, 0.157)	0.891	0.002	(−0.144, 0.149)	0.975^α^
Systolic bp (mmHG)	0.001	(−0.000, 0.002)	0.151	0.001	(−0.000, 0.002)	0.154	0.001	(−0.000, 0.002)	0.148^π^
Diastolic bp (mmHG)	0.003	(0.001, 0.004)	0.005	0.002	(0.000, 0.004)	0.041	0.002	(0.000, 0.004)	0.043^π^
**(b) Monozygotic twin pairs (*N*** = **62)**									
Waist circumference (cm)	0.001	(0.000, 0.003)	0.046	0.001	(−0.000, 0.003)	0.138			
HDL cholesterol (mmol/L)	−0.031	(−0.079, 0.018)	0.209	−0.004	(−0.062, 0.055)	0.901	−0.003	(−0.060, 0.054)	0.924 ^µ^
Triglycerides (mmol/L)^∞^	0.045	(0.003, 0.087)	0.037	0.040	(−0.010, 0.090)	0.115	0.036	(−0.014, 0.085)	0.152 ^µ^
Fasting glucose (mmol/L)^∞^	−0.026	(−0.235, 0.184)	0.807	−0.095	(−0.346, 0.156)	0.450	−0.099	(−0.351, 0.153)	0.433^α^
Systolic bp (mmHG)	0.001	(−0.001, 0.002)	0.371	0.000	(−0.001, 0.002)	0.483	0.000	(−0.001, 0.002)	0.478^π^
Diastolic bp (mmHG)	0.001	(−0.000, 0.003)	0.142	0.001	(−0.001, 0.003)	0.215	0.001	(−0.001, 0.004)	0.218^π^
**(c) Dizygotic twin pairs (*N*** = **37)**									
Waist circumference (cm)	0.002	(−0.000, 0.004)	0.058	0.001	(−0.001, 0.003)	0.292			
HDL cholesterol (mmol/L)	−0.103	(−0.191, −0.014)	0.025	−0.063	(−0.172, 0.046)	0.246	−0.083	(−0.188, 0.022)	0.116 ^µ^
Triglycerides (mmol/L)^∞^	0.051	(−0.011, 0.112)	0.103	0.044	(−0.010, 0.098)	0.107	0.044	(−0.008, 0.096)	0.096 ^µ^
Fasting glucose (mmol/L)^∞^	0.110	(−0.137, 0.357)	0.372	0.074	(−0.128, 0.276)	0.454	0.055	(−0.144, 0.253)	0.573^α^
Systolic bp (mmHG)	0.001	(−0.001, 0.004)	0.291	0.001	(−0.001, 0.003)	0.438	0.001	(−0.001, 0.003)	0.474^π^
Diastolic bp (mmHG)	0.005	(0.001, 0.009)	0.011	0.002	(−0.002, 0.006)	0.319	0.002	(−0.002, 0.006)	0.347^π^

*adjusted for smoking status, alcohol consumption, physical activity.

*HDL* high-density lipoprotein, *bp* blood pressure.

^µ^use of cholesterol lowering medications, ^α^use of blood glucose lowering medications, ^π^use of antihypertensives, ^∞^natural log transformation was performed due to skewed distribution of variable.

One sample excluded for extreme log-transformed fasting glucose (mmol/L) value of over 2.75.

*β* unstandardized regression coefficient, *CI* confidence interval.

The fixed-effects within-twin-pair regression analyses were conducted separately for the MZ and DZ twin pairs. Greater waist circumference (0.001, *p* = .046) and higher levels of triglycerides (0.045, *p* = .037) were associated with higher DunedinPACE estimates among the MZ twin pairs (Table 5b). Lower levels of HDL cholesterol were associated with higher DunedinPACE estimates among the DZ twin pairs (Table 5c). After further adjustments for lifestyle factors and medication, these associations were attenuated to nonsignificant levels.

### Replication analysis

The results of the replication analysis are presented as supplementary material (Supplement 3 and 4). The individual-level results derived from the YFS data (Supplement 3) were apparently similar to those derived from the two discovery cohorts, providing additional evidence that the association between MetS components and accelerated aging is independent of lifestyle factors considered in this study. The within-twin-pair results derived from the EH-Epi data (Supplement 4) suggest that genetics fully explain these associations not only for GrimAge but also for DunedinPACE. When using DunedinPACE, these results consistently showed weaker associations between MetS components and accelerated aging among MZ pairs, who share all their genetic variation, compared to DZ pairs, who share only 50%. This suggests that genetics is a significant confounding factor in this association.

## Discussion

This study investigated the association between MetS and epigenetic aging using two epigenetic clocks, GrimAge and DunedinPACE, in a study population that covered the adult lifespan. We employed a co-twin control study design, which is a powerful setting for controlling for genetic and familial confounding. The analyses were replicated in two cohorts from the same base population. To the best of our knowledge, this is the first study to report the association between MetS and novel epigenetic clock DunedinPACE, and/or considering the effects of genetic factors. Our pioneering findings suggest that MetS is associated with an accelerated pace of aging, as measured with DunedinPACE. This study demonstrates for the first time that the link between MetS and premature aging may be explained by genetics.

Our individual-level analyses revealed that epigenetic aging was accelerated among participants with MetS compared to those without MetS, irrespective of age and sex, which indicates that biological aging accelerates even before the onset of MetS-related chronic diseases. More precisely, epigenetic aging was accelerated by 2.6 years (GrimAge) and 0.12 years/calendar year (DunedinPACE) among participants with MetS compared to those without MetS. In addition, we found that an adverse profile in terms of individual MetS components was associated with accelerated aging, with waist circumference exhibiting the strongest association. Our results suggest that the association between accelerated aging and blood pressure is weaker compared to other MetS components. This may be explained by the relatively high number (11.2%) of participants taking antihypertensive medications. The results derived from the replication of the individual-level analyses in a large Finnish cohort study were apparently similar to those derived from the primary analyses, providing additional evidence that also high blood pressure is associated with accelerated aging. These findings are in line with previous research related to the association between MetS and epigenetic aging [8, 9, 15, 17–19, 38].

Based on our preliminary analyses using older generation clocks (data not shown) and prior literature, we opted to utilize epigenetic clocks, GrimAge and DunedinPACE, in our research. Previous studies using both older generation clocks and the GrimAge clock have suggested that GrimAge may best capture the DNAm changes associated with MetS and its components [9, 15]. It is noteworthy that the GrimAge clock is estimated based on seven DNAm surrogate markers, including leptin, which is associated with obesity [39], and may thus be more suitable than older generation clocks for estimating the association between age acceleration and metabolic features. However, in this study, we found stronger associations using DunedinPACE, which was trained to predict the pace of aging using longitudinal data based on physiological aging measures. Therefore, DunedinPACE can be a particularly good marker for assessing the effects of the age-related accumulation of risk factors for MetS on epigenetic aging.

The exact mechanisms through which MetS may accelerate aging remain unclear, but they are likely related to physiological responses to excess fat accumulation [6, 40]. Obesity is considered pro-aging because it is associated with increased oxidative stress and a proinflammatory state, which, in turn, enhance white blood cell turnover [41]. It has been suggested that excess reactive oxygen species may contribute to metabolic dysregulation, cell damage, and consequently aging [42]. Meanwhile, HDL cholesterol may modulate epigenetic aging processes due to its antiatherogenic effects, such as the removal of lipid deposits, which are accompanied by a reduction in cytotoxic effects [43]. Furthermore, HDL reduces oxidative stress in plasma and cellular compartments, and the signaling pathways in which it participates are interconnected with stress response and survival pathways [43]. The effects of oxidative stress on the metabolic dysregulation seen in MetS may be partially mediated by DNAm [44]. Although our study did not demonstrate a clear association between high blood pressure and epigenetic aging, it is well known that high blood pressure has numerous unfavorable effects on biological aging [45]. Several key mechanisms, such as inflammation and oxidative stress, are common to both biological aging and the development of high blood pressure.

In this study, we investigated the association between MetS components and different DNAm-based surrogate biomarkers for health-related plasma proteins to gain more precise information about the underlying mechanisms explaining the associations (see Supplementary Table 2). DNAm pack-years and DNAm plasminogen activator inhibitor, PAI-1, exhibited the strongest associations with MetS components. Smoking behavior is a significantly stronger predictor of DNAm age than other lifestyle factors, particularly when using the GrimAge algorithm for estimation [28, 46]. Furthermore, it is well documented that smoking is associated with metabolic abnormalities and increases the risk of MetS [47]. Our findings are in line with previous research [9, 19] supporting the role of DNAm PAI-1 as a major driver in the association of the GrimAge clock with MetS and its features. This is reasonable, as MetS-related increases in cytokines and free fatty acids increase the production of PAI-1 by the liver, which complements the overproduction of PAI-1 by adipose tissue [6].

Previous literature suggests that the rising prevalence of MetS can be explained by the obesogenic environment; therefore, it is urgent that researchers identify the epigenetic mechanisms mediating the environmental impact on MetS etiology to recommend appropriate therapies and intervention strategies [20]. In our study, in addition to age and sex, we were able to acknowledge the effects of smoking, alcohol consumption, and physical activity level, which are known to affect both DNAm and MetS etiology [6, 13, 47–49]. Interestingly, in the primary individual-level analyses of the study, these lifestyle factors explained the associations of MetS components with the GrimAge clock but not with DunedinPACE. However, in the replication of the individual-level analyses, the associations, which were stronger for DunedinPACE compared to GrimAge, were significant for both clocks independent of the influence of lifestyle factors. This provides additional evidence that the association between MetS components and accelerated aging is independent of the lifestyle factors considered in this study.

A major strength of the present study was its co-twin control design, which naturally controls for age, sex, year of birth, and familial factors (both genetic and nongenetic) that are shared within twin pairs and may affect both exposure and outcome. To the best of our knowledge, no previous study has acknowledged the effect of genetics in estimating the associations between epigenetic aging and MetS, even though genotype has an important influence on both MetS components and the epigenome [20]. Our approach allows to control for genetic confounding when assessing the association between MetS and epigenetic aging. The results derived from the primary within-twin-pair analyses suggested that of the MetS components, waist circumference and triglycerides are associated with the pace of aging irrespective of genetics. In contrast, the results indicated that the association between MetS and epigenetic aging measured using the GrimAge algorithm might be more influenced by genetic confounding. The within-twin-pair replication analyses indicated that genetics fully explain these associations for both GrimAge and DunedinPACE, providing additional evidence that genetics is a major confounder in the association between MetS and epigenetic aging.

In addition, one strength of our study was that the study population covered the age range from young adulthood to older individuals. In the primary analysis, we investigated the association between MetS and epigenetic aging among a study population aged 23–69 years. The results of the replication analyses, which included middle-aged (YFS study) or older (EH-Epi) participants representing the general population with a narrow age range, were similar to those of the primary analysis.

Because of the cross-sectional study design, we could not draw any causal conclusions. The findings of this study concern the Finnish population, which is representative of high-income populations of European ancestry. We cannot draw any firm conclusions on how our findings apply to different ethnic groups and socioeconomic circumstances. The lifestyle factors acknowledged in the study did not include, for example, the effects of diet or work-related stress factors, such as shift work, on the association between MetS and accelerated epigenetic aging. Given the complex and partially unclear pathogenesis of MetS and its components, it is reasonable to use blood-based clocks, which assess systemic age acceleration, in investigating the association between MetS and epigenetic age acceleration. However, it should be noted that we cannot draw conclusions regarding whether MetS is associated with tissue- or cell-specific age acceleration.

In conclusion, this study demonstrates for the first time that genetic factors play a significant role in influencing the relationship between MetS components and epigenetic aging. More research is needed to determine which lifestyle factors may potentially mediate or moderate the association between MetS and epigenetic aging. Understanding the effects of different MetS components on epigenetic aging may lead to interventions that can slow down the aging process and prevent age-related diseases.

## Availability of data and materials

All twin data used in this study can be found within the Biobank of the National Institute for Health and Welfare, Finland. All biobanked data are publicly available for use by qualified researchers following a standardized application procedure.

Pseudonymized ERMA datasets are available on reasonable request. To request the data, please contact Dr. Eija Laakkonen (eija.k.laakkonen@jyu.fi).

The YFS dataset comprises health-related participant data, which means that their use is restricted under the regulations on professional secrecy (Act on the Openness of Government Activities, 612/1999) and on sensitive personal data (Personal Data Act, 523/1999, implementing the EU data protection directive 95/46/EC). Due to these legal restrictions, the YFS data cannot be stored in public repositories or otherwise made publicly available. However, data access may be permitted on a case-by-case basis upon request. Data sharing outside the group is done in collaboration with the YFS group and requires a data-sharing agreement. Investigators can submit an expression of interest to the chairman of the publication committee (Prof. Mika Kähönen, Tampere University, Finland, or Prof. Terho Lehtimäki in relation to epigenetic and genetic data).

### Supplementary information

Supplementary Information
