# Supplementary material for: Metabolic syndrome and epigenetic aging: a twin study
Source: Int J Obes (Lond). 2024 Jan 25;48(6):778–87. doi: 10.1038/s41366-024-01466-x (PMC11129944; doi:10.1038/s41366-024-01466-x)
Supplement: Supplementary file 1 — Supplementary Information [file 41366_2024_1466_MOESM1_ESM.pdf]

Supplementary Table 1. Means and 95% confidence intervals of age acceleration in years (GrimAgeAA) and pace of aging in years/calendar year (DunedinPACE) by MetS status.

	**Participants without MetS** (*N*=212) mean (95% CI)	**Participants with MetS** (*N*=56) mean (95% CI)	p-value
**GrimAgeAA**	-0.549 (-1.053,-0.045)	2.078 (0.996,3.160)	3.5E-5
**DunedinPACE**	0.911 (0.896,0.927)	1.032 (1.002,1.063)	4.8E-11

CI, confidence interval; MetS, metabolic syndrome. Note: Adjusted for family relatedness, age, sex and age*sex interaction

Supplementary Table 2. The association between metabolic syndrome components and DNAm based surrogate biomarkers for health-related plasma proteins (components of GrimAge).

	**Waist circumference (cm)**			**HDL (mmol/L)**			**Triglycerides (mmol/L)** [∞]			**Fasting glucose (mmol/L)** [∞]			**Systolic bp (mmHG)**			**Diastolic bp (mmHG)**		
	β	b (95% CI)	p-value	β	b (95% CI)	p-value	β	b (95% CI)	p-value	β	b (95% CI)	p-value	β	b (95% CI)	p-value	β	b (95% CI)	p-value
**Pack-years**	0.142	0.010<br>(0.002, 0.018)	0.019	-0.209	-0.464<br>(-0.762, -0.166)	0.002	0.173	0.353<br>(0.132, 0.574)	0.002	0.140	1.129<br>(-0.078, 2.336)	0.067	-0.008	-0.000<br>(-0.008, 0.007)	0.901	0.045	0.004<br>(-0.008, 0.015)	0.539
**ADM**	0.065	0.004<br>(0.002, 0.007)	0.003	-0.032	-0.072<br>(-0.155, 0.011)	0.089	0.052	0.106<br>(0.036, 0.177)	0.003	0.033	0.262<br>(-0.013, 0.538)	0.062	0.003	0.000<br>(-0.002, 0.002)	0.896	0.035	0.003<br>(-0.000, 0.006)	0.066
**B2M**	0.035	0.002<br>(0.000, 0.004)	0.015	-0.026	-0.057<br>(-0.115, 0.000)	0.052	0.025	0.051<br>(0.001, 0.101)	0.048	0.010	0.080<br>(-0.126, 0.285)	0.446	0.003	0.000<br>(-0.001, 0.002)	0.852	0.014	0.001<br>(-0.001, 0.004)	0.407
**Cystatin C**	0.027	0.002<br>(0.000, 0.003)	0.008	-0.026	-0.057<br>(-0.107, -0.008)	0.023	0.018	0.036<br>(-0.005, 0.077)	0.085	0.010	0.074<br>(-0.099, 0.246)	0.399	-0.006	-0.000<br>(-0.002, 0.001)	0.599	0.013	0.001<br>(-0.001, 0.003)	0.324
**GDF15**	0.047	0.003<br>(0.001, 0.005)	4.3E-4	-0.025	-0.056<br>(-0.118, 0.005)	0.072	0.031	0.062<br>(0.013, 0.111)	0.013	0.035	0.258<br>(0.027, 0.489)	0.029	-0.006	-0.000<br>(-0.002, 0.001)	0.651	0.012	0.001<br>(-0.002, 0.004)	0.488
**Leptin**	0.019	0.001<br>(-0.000, 0.003)	0.154	0.020	0.044<br>(-0.010, 0.098)	0.110	0.027	0.054<br>(0.000, 0.108)	0.050	0.017	0.135<br>(-0.054, 0.323)	0.160	0.022	0.001<br>(-0.000, 0.002)	0.071	0.009	0.001<br>(-0.002, 0.003)	0.622
**PAI-1**	0.234	0.016<br>(0.010, 0.022)	3.8E-7	-0.148	-0.329<br>(-0.550, -0.107)	0.004	0.188	0.383<br>(0.207, 0.558)	2.9E-5	0.154	1.228<br>(0.436, 2.021)	0.003	0.060	0.003<br>(-0.002, 0.009)	0.276	0.134	0.011<br>(0.002, 0.019)	0.008
**TIMP-1**	0.028	0.002<br>(0.000, 0.003)	0.004	-0.017	-0.038<br>(-0.075, -0.002)	0.039	0.019	0.038<br>(0.005, 0.071)	0.023	0.012	0.098<br>(-0.033, 0.228)	0.142	-0.001	-0.000<br>(-0.001, 0.001)	0.946	0.011	0.001<br>(-0.001, 0.003)	0.279

[∞]Natural log transformation was performed due to skewed distribution of variable.
One sample excluded for extreme log-transformed fasting glucose (mmol/L) value of over 2.75.
ADM, adrenomedullin; B2M, beta-2 microglobulin; DNAm, DNA methylation; GDF15, growth differentiation factor 15; HDL, high-density lipoprotein; pb, blood pressure; PAI-1, plasminogen activation inhibitor 1; TIMP-I, tissue inhibitor metalloproteinase 1; β, standardized regression coefficient; b, unstandardized regression coefficient; CI, confidence interval. Note: Adjusted for family relatedness, age, sex and age*sex interaction

Supplement 1: Study populations

The Finnish Twin Cohort (FTC) includes the following three large cohort studies: FinnTwin12 (participants born in 1983–1987), FinnTwin16 (participants born in 1975–1979), and an older twin cohort (participants born before 1958) [1–3]. All three twin cohorts have been studied in an intensive longitudinal manner, and several subprojects have been conducted in laboratory conditions. The FTC participants included in our study comprised the following two age groups: young adults (age range from 23 to 42 years, n = 171, 48% female) and older adults (age range from 57 to 69 years, n = 51, 51% female). The participants had previously taken part in clinical in-person sub-studies involving tissue sampling and DNAm data generation, as well as in clinical data collection. All twins with successful GrimAge and DunedinPACE estimates and MetS determination (n = 222) were included in the current study, with 62 complete monozygotic (MZ) twin pairs and 37 complete dizygotic (DZ) twin pairs. Some of the twin pairs in the dataset were MZ twin pairs selected for sub-studies and a more detailed laboratory analysis based on a discordance in body mass index (within-pair difference over 3 kg/m^2) [4]. In addition, this study included middle-aged female participants (age range from 48 to 55 years, n = 46) from the Estrogenic Regulation of Muscle Apoptosis (ERMA) study (https://doi.org/10.17011/jyx/dataset/83491) [5]. The ERMA study was designed to reveal how hormonal differences during the menopausal stages affect the physiological and psychological functioning of middle-aged women.

The Young Finns Study (YFS) is a long-term epidemiological study that aims to investigate the risk factors for cardiovascular disease from childhood to adulthood. In total, 3,596 children aged 3–18 years participated in the baseline examination in 1980. The participants have been followed up regularly. The YFS participants who had provided both whole blood samples for subsequent DNAm analyses and phenotypic data in 2011 were included in the replication dataset of our study if their MetS status had been determined and if both GrimAge and

DunedinPACE estimates had been successfully calculated. The dataset consisted of 1 564 participants (55% female) aged 34–49 years. The study design and methods for the YFS have been described in detail previously [6,7].

Essential Hypertension Epigenetics Study (EH-Epi)

Twins were selected based on responses to items on blood pressure and hypertension in the fourth survey of the FTC in 2011–2012; twin pairs with a difference in blood pressure were targeted, as previously described in detail [3]. The twins came in for 1 day of measurement of blood pressure, completed interviews and questionnaires and provided a fasting blood sample for biochemical measures, and samples for omics. In addition, weight, height, and waist and hip circumference were measured [8].

Metabolomic data for 434 participants were collected with nuclear magnetic resonance spectroscopy. The proportion of individuals with methylation (Illumina 450k) data was lower ($n = 360$). All twins, aged 56–69 years, with successful GrimAge and DunedinPACE estimates and MetS determination ($n = 293$, 61% female) were included in the current study, with 81 complete MZ twin pairs and 49 complete DZ twin pairs.

References

1. Kaidesoja M, Aaltonen S, Bogl LH, et al. FinnTwin16: A Longitudinal Study from Age 16 of a Population-Based Finnish Twin Cohort. *Twin Res Hum Genet* 2019; 22: 530-39.
2. Rose RJ, Salvatore JE, Aaltonen S, et al. FinnTwin12 Cohort: An Updated Review. *Twin Res Hum Gen* 2019; 22: 302-11.
3. Kaprio J, Bollepalli S, Buchwald J, et al. The Older Finnish Twin Cohort — 45 Years of Follow-up. *Twin Res Hum Gen* 2019; 22: 240-54.

4. Naukkarinen J, Rissanen A, Kaprio J, Pietiläinen KH. Causes and consequences of obesity: the contribution of recent twin studies. *Int J Obes* 2012; 36: 1017-24.

5. Kovanen V, Aukee P, Kokko K, et al. Design and protocol of Estrogenic Regulation of Muscle Apoptosis (ERMA) study with 47 to 55-year-old women's cohort: novel results show menopause-related differences in blood count. *Menopause* 2018; 25: 1020-32.

6. Raitakari OT, Juonala M, Ronnemaa T, et al. Cohort Profile: The Cardiovascular Risk in Young Finns Study. *Int J Epidemiol* 2008; 37: 1220-26.

7. Juonala M, Magnussen CG, Berenson GS, et al. Childhood Adiposity, Adult Adiposity, and Cardiovascular Risk Factors. *N Engl J Med* 2011; 365: 1876-85.

8. Tuomela J, Kaprio J, Sipilä PN, et al. Accuracy of self-reported anthropometric measures—Findings from the Finnish Twin Study. *Obes Res Clin Pract* 2019;13: 522–528

Supplement 2: Research ethics

The Finnish Twin Cohort (FTC) data collection and analysis were approved by the Ethics Committee of the Helsinki University Central Hospital (Dnro 249/E5/01, 270/13/03/01/2008, 154/13/03/00/2011), and the Estrogenic Regulation of Muscle Apoptosis (ERMA) study was approved by the Ethics Committee of the Central Finland Health Care District in 2014 (K-SSHP Dnro 8U/2014). Written informed consent was provided by the participants before the beginning of the studies.

The Young Finns Study (YFS) was approved by the Ethics Committee of the Hospital District of Southwest Finland (ETMK: 68/1801/2017) and the Regional Ethics Committee of the Expert Responsibility Area of Tampere University Hospital, Helsinki University Hospital Ethics Committee of Medicine, the Research Ethics Committee of the Northern Savo Hospital District, and the Ethics Committee of the Northern Ostrobothnia Hospital District. The study protocol for each study phase followed the World Health Organization proposal. Written informed consent was obtained from all the examined participants, and the study was conducted in accordance with the Helsinki Declaration. For previous follow-ups of the YFS, parental and/or legal guardian consent was obtained for participants under the age of 18 years.

Essential Hypertension Epigenetics Study (EH-Epi) was approved by the Institutional Ethics Board of the Hospital District of Helsinki and Uusimaa, Finland (ID 154/13/03/00/11) and the Institutional Review Board of Augusta University. Omics datasets were obtained from within the FTC for up to 444 twins, and all applicable written and informed consent was obtained in relation to the data generated or used for analysis.

Supplement 3: The results of the replication analysis: Young Finns Study

Table 1. Descriptive characteristics of the participants of the replication data (Young Finns Study) by MetS status: Presentation of MetS component characteristics separately for all, female, and male participants.

Characteristic	Participants without MetS (*N*=1213)	Participants with MetS (*N*=351)
Sex, N (%) of participants		
Female	708 (58.4)	146 (41.6)
Male	505 (41.6)	205 (58.4)
Age, mean (SD) range, years	41.7 (5.0)	43.3 (4.6)
Cigarette smoking, N (%) of participants		
Never smokers	643 (53.9)	163 (47.8)
Former smokers	302 (25.3)	92 (27.0)
Current smokers	247 (20.7)	86 (25.2)
Alcohol, mean (SD), drinks per week[∞]	5.2 (7.5)	7.1 (10.6)
Level of physical activity, N (%) of participants		
Low	147 (12.3)	66 (19.6)
Medium	330 (27.7)	108 (32.0)
High	716 (60.0)	163 (48.4)
Body mass index, mean (SD), kg/m^2	25.1 (3.9)	31.6 (5.2)
Components of MetS, mean (SD)		
Waist Circumference (cm)		
All	87.5 (11.4)	106.9 (12.8)
Female	84.0 (11.2)	103.5 (12.3)
Male	92.5 (9.7)	109.3 (12.7)
HDL cholesterol (mmol/l)		
All	1.4 (0.3)	1.1 (0.3)
Female	1.5 (0.3)	1.2 (0.3)
Male	1.3 (0.3)	1.0 (0.2)
Triglycerides (mmol/l)		
All	1.1 (0.5)	2.1 (1.1)
Female	0.9 (0.4)	1.8 (1.0)
Male	1.2 (0.6)	2.3 (1.2)
Fasting glucose (mmol/l)		
All	5.2 (0.7)	5.9 (1.2)
Female	5.1 (0.8)	5.8 (1.1)
Male	5.4 (0.5)	6.0 (1.3)
Blood pressure (mmHg)		
Systolic		
All	123.3 (13.7)	135.8 (14.3)
Female	118.7 (13.0)	130.6 (12.6)
Male	129.7 (12.1)	139.5 (14.3)
Diastolic		
All	75.7 (9.1)	86.0 (10.0)
Female	74.3 (8.8)	84.0 (8.9)
Male	77.7 (9.0)	87.3 (10.4)
Medication, N (%) of participants		
Cholesterol	31 (2.6)	31 (9.2)
Hypertension	64 (5.4)	90 (26.6)
Glucose	6 (0.5)	20 (5.9)

[∞] one drink = 12 g/100% alcohol

DNAm, DNA methylation; HDL, high-density lipoprotein; MetS, metabolic syndrome

Table 2. The association of between metabolic syndrome components and age acceleration in years (GrimAgeAA) in replication data (Young Finns Study).

	**Model 1**			**Model 2**			**Model 2 + medication**		
	β	b (95% CI)	p-value	β	b (95% CI)	p-value	β	b (95% CI)	p-value
**Waist circumference (cm)**	0.150	0.038 (0.025, 0.051)	1.3E-8	0.118	0.030 (0.019, 0.042)	1.8E-7			
**HDL cholesterol (mmol/L)**	-0.048	-0.522 (-1.081, 0.037)	0.067	-0.052	-0.571 (-1.053, -0.089)	0.020	-0.048	-0.524 (-1.010, -0.037)	0.035[µ]
**Triglycerides (mmol/L)**[∞]	0.148	1.060 (0.702, 1.417)	7.2E-9	0.087	0.628 (0.317, 0.939)	7.8E-5	0.084	0.608 (0.294, 0.921)	1.5E-4[µ]
**Fasting glucose (mmol/L)** [∞]	0.099	3.153 (1.547, 4.759)	1.2E-4	0.065	2.060 (0.685, 3.434)	0.003	0.060	1.944 (0.462, 3.426)	0.010[α]
**Systolic bp (mmHG)**	0.062	0.015 (0.002, 0.028)	0.023	0.066	0.016 (0.005, 0.028)	0.004	0.054	0.013 (0.002, 0.025)	0.023[π]
**Diastolic bp (mmHG)**	0.065	0.023 (0.005, 0.041)	0.011	0.067	0.024 (0.009, 0.040)	0.002	0.055	0.020 (0.004, 0.036)	0.015[π]

Model 1 adjusted for age and sex, (age*sex)
Model 2 adjusted for age, sex, (age*sex), smoking status, alcohol consumption, and physical activity
HDL, high-density lipoprotein; bp, blood pressure
[µ]use of cholesterol lowering medications, [α]use of blood glucose lowering medications, [π]use of antihypertensives, [∞]natural log transformation was performed due to skewed distribution of variable
Three samples excluded for extreme log-transformed fasting glucose (mmol/L) value of over 2.70.
β, standardized regression coefficient; b, unstandardized regression coefficient; CI, confidence interval.

Table 3. The association between metabolic syndrome components and pace of aging in years/calendar year (DunedinPACE) in replication data (Young Finns Study).

	Model 1			Model 2			Model 2 + medication		
	β	b (95% CI)	p-value	β	b (95% CI)	p-value	β	b (95% CI)	p-value
**Waist circumference (cm)**	0.340	0.002 (0.002, 0.003)	5.1E-39	0.307	0.002 (0.002, 0.002)	2.4E-34			
**HDL cholesterol (mmol/L)**	-0.179	-0.052 (-0.067, -0.037)	7.1E-12	-0.170	-0.049 (-0.064, -0.035)	2.0E-11	-0.168	-0.049 (-0.063, -0.034)	4.7E-11$^{\mu}$
**Triglycerides (mmol/L)** $^{\infty}$	0.238	0.045 (0.036, 0.054)	1.6E-20	0.190	0.036 (0.027, 0.045)	2.1E-14	0.190	0.036 (0.027, 0.045)	3.6E-14$^{\mu}$
**Fasting glucose (mmol/L)** $^{\infty}$	0.150	0.126 (0.083, 0.168)	8.3E-9	0.128	0.107 (0.066, 0.148)	3.2E-7	0.118	0.100 (0.056, 0.144)	9.9E-6$^{\alpha}$
**Systolic bp (mmHG)**	0.118	0.001 (0.000, 0.001)	1.6E-5	0.132	0.001 (0.001, 0.001)	4.7E-7	0.117	0.001 (0.000, 0.001)	1.1E-5$^{\pi}$
**Diastolic bp (mmHG)**	0.145	0.001 (0.001, 0.002)	2.2E-8	0.148	0.001 (0.001, 0.002)	2.4E-9	0.135	0.001 (0.001, 0.002)	8.9E-8$^{\pi}$

Model 1 adjusted for age and sex, (age*sex)

Model 2 adjusted for age, sex, (age*sex), smoking status, alcohol consumption, and physical activity

HDL, high-density lipoprotein; pb, blood pressure

$^{\mu}$use of cholesterol lowering medications, $^{\alpha}$use of blood glucose lowering medications, $^{\pi}$use of antihypertensives, $^{\infty}$natural log transformation was performed due to skewed distribution of variable

Three samples excluded for extreme log-transformed fasting glucose (mmol/L) value of over 2.70.

β, standardized regression coefficient; b, unstandardized regression coefficient; CI, confidence interval.

Table 4. Means and 95% confidence intervals of age acceleration in years (GrimAgeAA) and pace of aging in years/calendar year (DunedinPACE) according to the status of metabolic syndrome in replication data (Young Finns Study).

	**Participants without MetS** (N=1213) mean (95% CI)	**Participants with MetS** (N=351) mean (95% CI)	p-value
**GrimAgeAA**	-0.238 (-0.435,-0.041)	0.822 (0.452,1.192)	9.3E-7
**DunedinPACE**	1.004 (0.999,1.009)	1.062 (1.053,1.072)	1.5E-24

CI, confidence interval; MetS, metabolic syndrome. Note: Adjusted for age, sex, and age*sex interaction

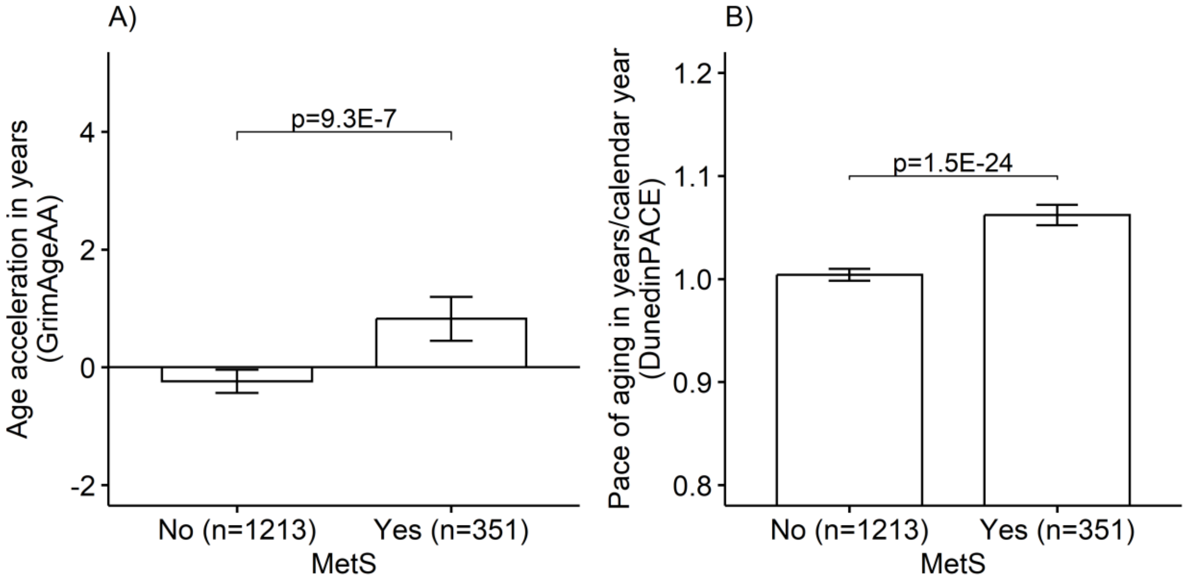

Figure 1. Means and confidence intervals of age acceleration (GrimAgeAA) and pace of aging (DunedinPACE) according to the status of metabolic syndrome (MetS) in replication data (Young Finns Study). Note: Adjusted for age, sex, and age*sex interaction.

Supplement 4: The results of the replication analysis: Essential Hypertension Epigenetics Study

Table 1. Descriptive characteristics of the participants of the replication data (Essential Hypertension Epigenetics Study) by MetS status: Presentation of MetS component characteristics separately for all, female, and male participants.

**Characteristic**	**Participants without MetS** (*N*=198)	**Participants with MetS** (*N*=95)
Sex, N (%) of participants		
Female	126 (63.6)	53 (55.8)
Male	72 (36.4)	42 (44.2)
Age, mean (SD) range, years	61.9 (3.7)	61.6 (3.7)
Cigarette smoking, N (%) of participants		
Never smokers	104 (52.5)	37 (38.9)
Former smokers	67 (33.8)	36 (37.9)
Current smokers	27 (13.6)	22 (23.2)
Alcohol, mean (SD), drinks per week[∞]	4.3 (6.7)	4.8 (5.9)
Level of physical activity, N (%) of participants		
Low	30 (15.3)	21 (22.1)
Medium	89 (45.4)	39 (41.1)
High	77 (39.3)	35 (36.8)
Body mass index, mean (SD), kg/m^2	26.0 (4.5)	28.7 (5.0)
Components of MetS, mean (SD)		
Waist Circumference (cm)		
All	91.4 (12.9)	99.9 (14.3)
Female	87.3 (12.2)	95.2 (13.1)
Male	98.8 (10.8)	105.9 (13.6)
HDL cholesterol (mmol/l)		
All	1.6 (0.4)	1.3 (0.4)
Female	1.8 (0.3)	1.5 (0.4)
Male	1.4 (0.3)	1.1 (0.3)
Triglycerides (mmol/l)		
All	1.1 (0.3)	1.5 (0.7)
Female	1.0 (0.3)	1.3 (0.6)
Male	1.1 (0.3)	1.6 (0.8)
Fasting glucose (mmol/l)		
All	4.4 (0.4)	4.8 (1.0)
Female	4.3 (0.4)	4.6 (1.0)
Male	4.5 (0.4)	5.0 (0.9)
Blood pressure (mmHg)		
Systolic		
All	143.6 (16.9)	144.7 (16.4)
Female	141.2 (17.7)	140.0 (16.0)
Male	147.8 (14.8)	150.5 (15.1)
Diastolic		
All	84.5 (9.5)	84.5 (10.9)
Female	83.3 (9.4)	82.0 (10.3)
Male	86.5 (9.5)	87.6 (10.9)
Medication, N (%) of participants		
Cholesterol	1 (0.5)	60 (63.2)
Hypertension	61 (30.8)	62 (65.3)
Glucose	0 (0)	14 (14.7)

[∞] one drink = 12 g/100% alcohol

DNAm, DNA methylation; HDL, high-density lipoprotein; MetS, metabolic syndrome

Table 2. The association between metabolic syndrome components and age acceleration in years (GrimAgeAA) in replication data (Essential Hypertension Epigenetics Study): Within-twin-pair analyses, (a) all pairs, (b) monozygotic twin pairs, and (c) dizygotic twin pairs.

	Base model			Adjusted model*			Adjusted model* + medication		
	β	95% CI	p-value	β	95% CI	p-value	β	95% CI	p-value
**a) All pairs (*N*=130*)***									
Waist circumference (cm)	0.058	0.016, 0.100	0.007	0.057	0.024, 0.089	0.001			
HDL cholesterol (mmol/L)	-0.734	-2.427, 0.959	0.393	-1.306	-2.611, -0.002	0.050	-1.281	-2.592, 0.030	0.055$^{\mu}$
Triglycerides (mmol/L) $^{\infty}$	0.489	-0.863, 1.841	0.476	0.960	-0.083, 2.003	0.071	1.038	-0.017, 2.092	0.054$^{\mu}$
Fasting glucose (mmol/L) $^{\infty}$	6.096	2.144, 10.048	0.003	4.971	1.910, 8.032	0.002	5.075	1.919, 8.231	0.002$^{\alpha}$
Systolic bp (mmHG)	0.016	-0.017, 0.048	0.340	0.015	-0.011, 0.040	0.261	0.020	-0.005, 0.045	0.121$^{\pi}$
Diastolic bp (mmHG)	0.009	-0.041, 0.059	0.732	0.026	-0.013, 0.066	0.118	0.033	-0.006, 0.072	0.094$^{\pi}$
**b) Monozygotic twin pairs (*N*=81)**									
Waist circumference (cm)	0.009	-0.043, 0.060	0.732	0.029	-0.012, 0.071	0.159			
HDL cholesterol (mmol/L)	-0.055	-1.935, 1.824	0.953	-0.748	-2.240, 0.744	0.321	-0.724	-2.221, 0.773	0.338$^{\mu}$
Triglycerides (mmol/L) $^{\infty}$	0.816	-0.642, 2.275	0.269	0.650	-0.465, 1.764	0.249	0.570	-0.579, 1.720	0.326$^{\mu}$
Fasting glucose (mmol/L) $^{\infty}$	2.867	-2.369, 8.102	0.279	2.669	-1.662, 7.000	0.223	2.578	-2.445, 7.601	0.310$^{\alpha}$
Systolic bp (mmHG)	0.011	-0.022, 0.044	0.505	0.024	-0.003, 0.050	0.075	0.022	-0.007, 0.050	0.131$^{\pi}$
Diastolic bp (mmHG)	0.011	-0.042, 0.063	0.686	0.036	-0.005, 0.077	0.087	0.032	-0.010, 0.075	0.129$^{\pi}$
**c) Dizygotic twin pairs (*N*=49)**									
Waist circumference (cm)	0.093	0.023, 0.163	0.011	0.071	0.016, 0.126	0.013			
HDL cholesterol (mmol/L)	-1.349	-4.457, 1.759	0.387	-2.148	-4.582, 0.287	0.082	-1.983	-4.500, 0.533	0.119$^{\mu}$
Triglycerides (mmol/L) $^{\infty}$	0.168	-2.379, 2.715	0.895	1.803	-0.334, 3.939	0.096	1.817	-0.321, 3.956	0.094$^{\mu}$
Fasting glucose (mmol/L) $^{\infty}$	7.839	1.449, 14.230	0.017	6.135	1.134, 11.135	0.017	6.111	1.030, 11.192	0.020$^{\alpha}$
Systolic bp (mmHG)	0.021	-0.043, 0.085	0.513	-0.008	-0.064, 0.049	0.786	-0.003	-0.056, 0.049	0.903$^{\pi}$
Diastolic bp (mmHG)	0.006	-0.091, 0.104	0.895	0.008	-0.072, 0.088	0.844	0.015	-0.059, 0.089	0.684$^{\pi}$

*adjusted for smoking status, alcohol consumption, physical activity

HDL, high-density lipoprotein; bp, blood pressure

$^{\mu}$use of cholesterol lowering medications, $^{\alpha}$use of blood glucose lowering medications, $^{\pi}$use of antihypertensives, $^{\infty}$natural log transformation was performed due to skewed distribution of variable

β, unstandardized regression coefficient; CI, confidence interval.

Table 3. The association between metabolic syndrome components and the pace of aging in years/calendar year (DunedinPACE) in replication data (Essential Hypertension Epigenetics Study): Within-twin-pair analyses, (a) all pairs, (b) monozygotic twin pairs, and (c) dizygotic twin pairs.

	**Base model**			**Adjusted model***			**Adjusted model* + medication**		
	β	95% CI	p-value	β	95% CI	p-value	β	95% CI	p-value
**a) All pairs (*N*=130)**									
Waist circumference (cm)	0.003	0.002, 0.004	4.0E-6	0.003	0.002, 0.004	7.0E-7			
HDL cholesterol (mmol/L)	-0.081	-0.132, -0.030	0.002	-0.101	-0.149, -0.054	4.6E-5	-0.097	-0.143, -0.051	6.0E-5[µ]
Triglycerides (mmol/L) [∞]	0.046	0.005, 0.088	0.029	0.058	0.019, 0.098	0.004	0.068	0.031, 0.106	4.7E-4[µ]
Fasting glucose (mmol/L) [∞]	0.176	0.052, 0.299	0.006	0.144	0.025, 0.264	0.018	0.134	0.011, 0.257	0.033[α]
Systolic bp (mmHG)	0.001	-0.000, 0.002	0.270	0.001	-0.000, 0.002	0.206	0.001	-0.000, 0.002	0.101[π]
Diastolic bp (mmHG)	0.001	-0.001, 0.003	0.199	0.001	-0.000, 0.003	0.061	0.002	0.000, 0.003	0.027[π]
**b) Monozygotic twin pairs (*N*=81)**									
Waist circumference (cm)	0.001	-0.000, 0.003	0.139	0.002	-0.000, 0.003	0.062			
HDL cholesterol (mmol/L)	-0.045	-0.109, 0.019	0.166	-0.067	-0.129, -0.006	0.033	-0.069	-0.130, -0.008	0.027[µ]
Triglycerides (mmol/L) [∞]	0.025	-0.026, 0.075	0.334	0.019	-0.028, 0.066	0.424	0.029	-0.019, 0.077	0.236[µ]
Fasting glucose (mmol/L) [∞]	0.099	-0.082, 0.279	0.279	0.076	-0.108, 0.259	0.415	0.148	-0.063, 0.358	0.166[α]
Systolic bp (mmHG)	0.001	-0.000, 0.002	0.201	0.001	-0.000, 0.002	0.058	0.001	-0.000, 0.002	0.080[π]
Diastolic bp (mmHG)	0.001	-0.001, 0.003	0.320	0.001	-0.000, 0.003	0.100	0.001	-0.000, 0.003	0.129[π]
**c) Dizygotic twin pairs (*N*=49*)***									
Waist circumference (cm)	0.004	0.002, 0.006	2.7E-5	0.004	0.002, 0.006	2.4E-5			
HDL cholesterol (mmol/L)	-0.114	-0.199, -0.028	0.010	-0.142	-0.224, -0.061	0.001	-0.126	-0.207, -0.045	0.003[µ]
Triglycerides (mmol/L) [∞]	0.068	-0.004, 0.139	0.065	0.125	0.054, 0.197	0.001	0.127	0.061, 0.193	3.9E-4[µ]
Fasting glucose (mmol/L) [∞]	0.217	0.029, 0.405	0.025	0.197	0.009, 0.384	0.040	0.185	0.000, 0.369	0.050[α]
Systolic bp (mmHG)	0.000	-0.002, 0.002	0.709	0.000	-0.002, 0.002	0.964	0.000	-0.002, 0.002	0.818[π]
Diastolic bp (mmHG)	0.001	-0.002, 0.004	0.424	0.001	-0.001, 0.004	0.310	0.002	-0.001, 0.004	0.187[π]

*adjusted for smoking status, alcohol consumption, physical activity

HDL, high-density lipoprotein; bp, blood pressure

[µ]use of cholesterol lowering medications, [α]use of blood glucose lowering medications, [π]use of antihypertensives, [∞]natural log transformation was performed due to skewed distribution of variable

β, unstandardized regression coefficient; CI, confidence interval.
